# Monthly Summary

**Published:** 1898-09

**Authors:** 


### Monthly Summary.

THE PATENT BILL.—Congress adjourned without action on the bill regulating patents, of which we spoke last month. In a letter received from Dr. Ottolengui recently, he says:

"In this connection I take pleasure in informing you that although Congress has adjourned without the passage of the bill, nevertheless a great deal has been accomplished. The present situation is, the bill has been introduced in both houses of Congress and referred to the proper committees. The Senate committee accorded us a hearing, and we are assured that as soon as the bill is recommended by the House committee it will be favorably reported by the Senate committee.

"The bill did not reach the House committee until very late in the session, and it was impossible to get a quorum after the reference; thanks, however, to the letters which reached the committee from their constituents, I am in a position to say that practically the majority of this committee also favored the bill. All that will be necessary in the autumn will be to give some evidence to our representatives in Congress, when the matter is taken up again, that the dentists of the country, especially the organized societies of dentists, really desire this legislation, and there will be no obstacle in the way of its passage."

In the pressure of matters of more general interest before Congress, the gentlemen having this matter in charge achieved gratifying results. It is to be hoped that another effort in the fall will result successfully.—"Indiana Dental Journal."

---

DESTRUCTION OF PULP AND GOLD CROWN BY MERCURIC ACTION WITHIN AMALGAM.—I have been greatly interested in the result produced by an amalgam filling inserted in a lower third molar, which was covered with a gold crown. The entire buccal surface of a right lower third molar was filled in March, 1894. The cavity extended below the gum margin and was not very deep. A few months after its insertion the patient returned and complained of a peculiar and very unpleasant taste about this particular tooth. It became very offensive. I attributed the annoyance to oxidation or an acid condition of the fluids of the mouth affecting the amalgam. I suggested that crowning the tooth with gold would no doubt prevent any further inconvenience, thinking that more convenient, as well as more permanent. The patient permitted the operation as suggested, and it was entirely successful. The unpleasantness became entirely eliminated. This patient returned a few week ago, being absent about four years—quite a long time without consulting a dentist. Her visit was for the purpose of having her teeth examined on account of some slight inconvenience. Imagine my amazement when I beheld the gold crown entirely disintegrated and porosity everywhere noticeable, and the slightest effort removed it. This was surely the result of a physical and possibly a physiological action of mercury. I have always felt that there are idiosyncrasies whereby amalgam or mercurial medicaments are contraindicated, and I believe that this experiment will materially assist to bear out this hardly settled question. It has taken about four years for this change to occur—pulp is dead, incipient decay around a part of amalgam filling.

I have reprepared cavity, cleansed and antiseptically treated canals, filled them up with powdered asbestos saturated with a 50 per cent. solution of silver nitrat; filled tooth with gutta-percha, and made new gold crown, 21k., and set it in. Saw patient recently, and she is enjoying life from a masticatory standpoint, and I thinking, consciously and scientifically, which is to blame, McLean or mercury? Some stomatological brother will confer a favor if he will explain.—"W. T. McLean, in Ohio Dental Journal."

---

TIN.—Dr. J. Y. Crawford is a strong advocate of tin to be used on the floor and walls of teeth of weak structure, filling with cement and afterwards removing the cement and replacing with gold.

It is a source of great satisfaction to notice how well preserved and improved are the walls of a cavity years after this treatment has been used. To those who have been reluctant in the use of tin in the anterior teeth of children, fear no longer. Though there may be a dark appearance while the tin is retained, when the time comes, say at maturity, these fillings can be replaced with gold, and there will not be the slightest discoloration from the oxid of tin; but should there be, is it not better to have a good tooth which is a little dark than a crown or bridge, or possibly an artificial denture?

To prevent discoloration of the cavity walls, use varnish and dry hot air. This subject is getting old, but it should be insisted upon until its merits are overcome by better methods. —" W. H. Weaver, in Dental Weekly."

---

SHRINKAGE OF AMALGAMS.—Thomas Fletcher, F. C. S. —The peculiarity of amalgams in which a small quantity of mercury is used, to which Mr. Kirby calls attention—viz.; a slight shrinkage round the upper edge—has occupied my attention for some time, and a series of careful experiments proves undoubtedly that it is caused by the middle of the plug being condensed without proper attention being paid to the edges. If the edges are carefully burnished down when the amalgam is partially hardened, this shrinkage does not take place unless an excess of mercury is used, which is forced to the surface by pressure, causing an unequal mixture, having different properties to the body of the plug.—*British Journal of Dental Science.*

---

GETTING THE BITE.—In edentulous cases, or where there are very few teeth left in the mouth, it is often very difficult to get a correct bite. Get the patient to throw his head well back and ask him to swallow. He is then bound to articulate the lower correctly with the upper. Repeat the process two or three times, and it will be seen that the patient will bite the same every time. The important point is to get the patient's head well back.—" F. Mackenzie, in British Journal of Dental Science.

